# Supplementary figures and images for: Contribution of parasite and host genotype to immunopathology of schistosome infections
Source: Parasit Vectors. 2024 May 7;17:203. doi: 10.1186/s13071-024-06286-6 (PMC11073996; doi:10.1186/s13071-024-06286-6)

**A**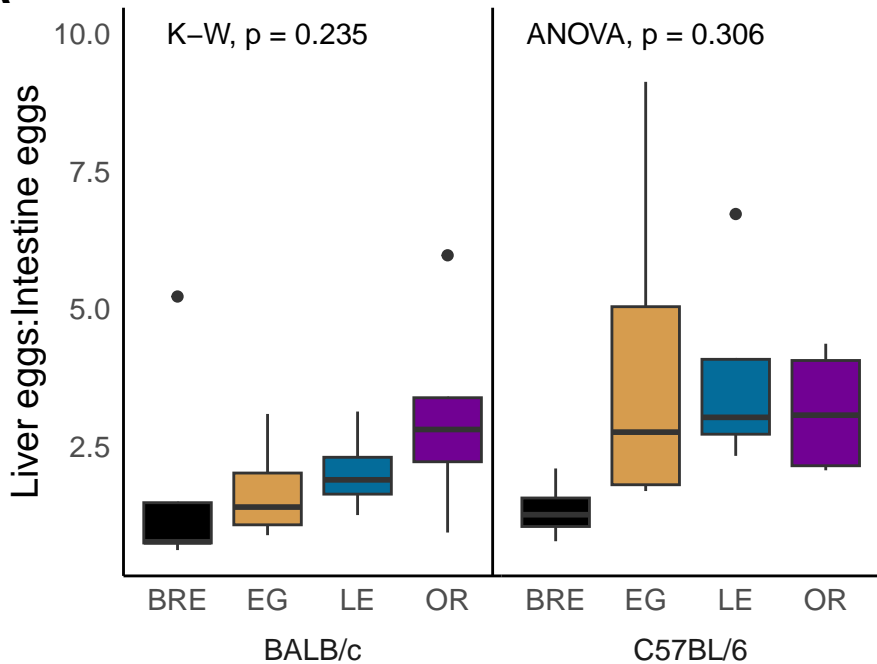

Supplement: Supplementary file 2 — Additional file 2: Figure S1. Box plot showing the ratio of liver eggs to intestine eggs in schistosome-infected BALB/c (left) and C57BL/6 mice (right). Statistical comparison between parasite populations done for each host with Kruskal-Wallis (K-W) or ANOVA (BALB/c: Kruskal-Wallis; H = 4.26, df = 3, P = 0.235; C57BL/6: ANOVA; F(3, 12) = 1.34, P = 0.306). Differences between hosts were not significant (Wilcox test; W = 97, P = 0.109). [file 13071_2024_6286_MOESM2_ESM.pdf]

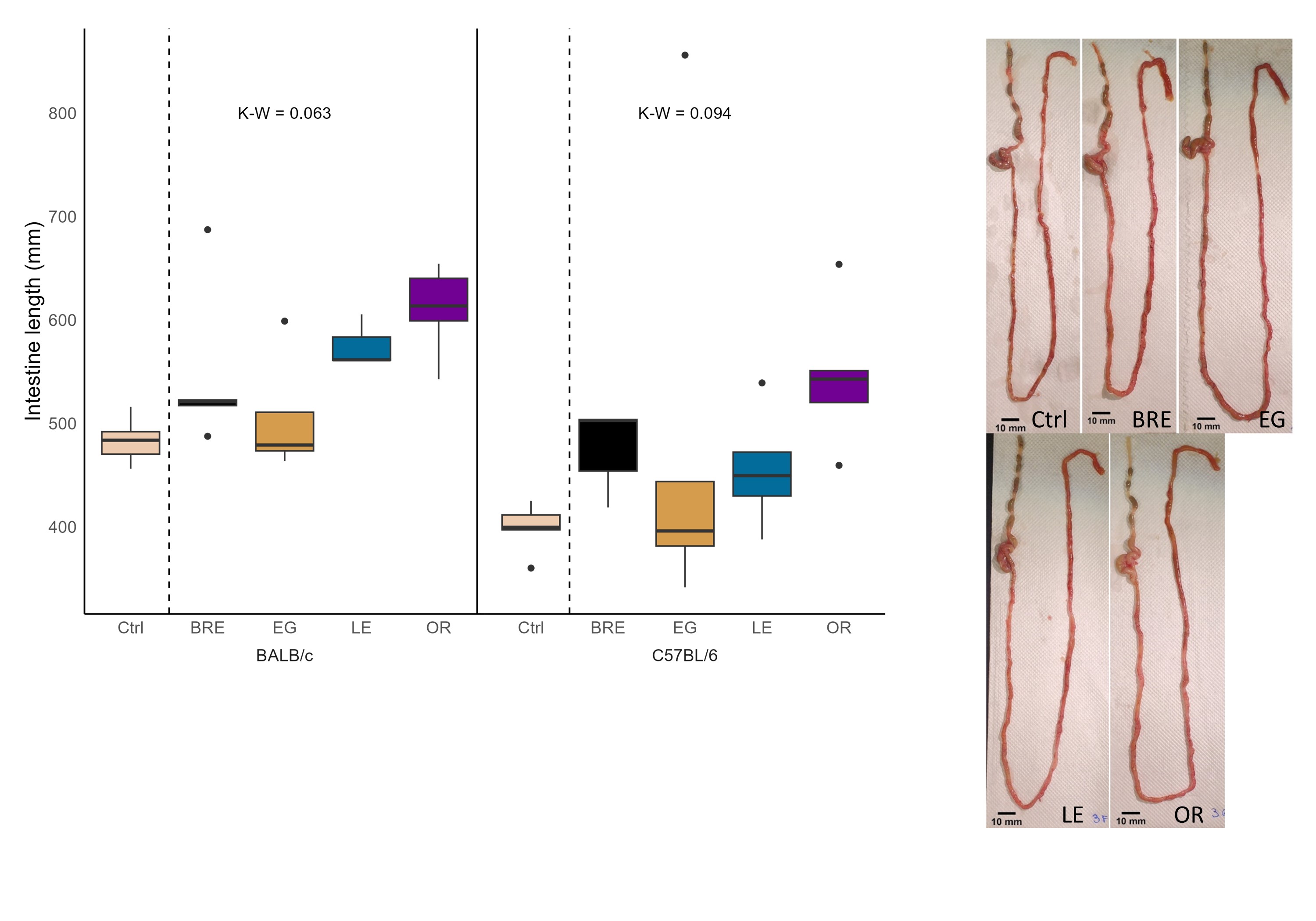

Supplement: Supplementary file 3 — Additional file 3: Figure S2. Intestine length of schistosome-infected BALB/c and C57BL/6 mice. Box plot showing intestine length in BALB/c (left) and C57BL/6 mice (right) infected with the four parasite populations or uninfected controls. Photos (right) show representative intestine samples from C57BL/6 mice infected with the four parasite populations and the control group for reference. Kruskal-Wallis to identify comparisons between parasite populations (BALB/c: H = 7.29, P = 0.063; C57BL/6: H = 6.38, P = 0.094) and Wilcoxon test to compare hosts, which were not significant. [file 13071_2024_6286_MOESM3_ESM.jpg]

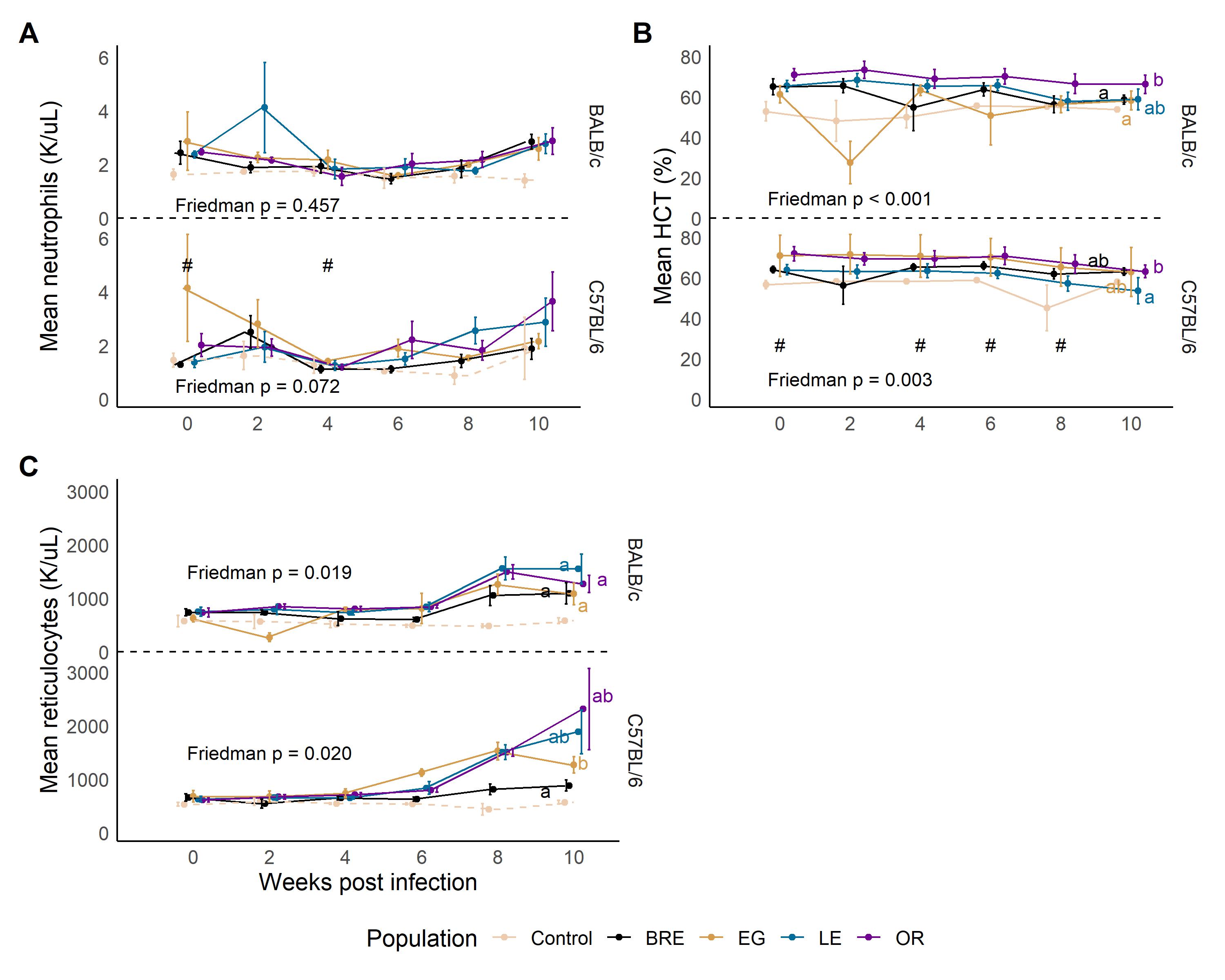

Supplement: Supplementary file 4 — Additional file 4: Figure S3. Neutrophil and reticulocyte levels and mean hematocrit (HCT) output. Longitudinal plots showing select CBC levels in BALB/c (top) and C57BL/6 mice (bottom) infected with the four parasite populations (solid lines) or uninfected control (dashed lines). A Neutrophil levels (Wilcoxon test to compare hosts: Baseline: W = 264, P = 0.042; Week 4: W = 275, P = 0.034). B Hematocrit (HCT) (Friedman; BALB/c: χ2 = 16.4, df = 3, P < 0.001; C57BL/6: χ2 = 13.8, df = 3, P = 0.003). Plot shows significant differences between hosts at baseline, and weeks 4, 6 and 8 (Wilcoxon test; Baseline: W = 101, P = 0.032; Week 4: W = 84, P = 0.016; Week 6: W = 77, P = 0.016; Week 8: W = 94.5, P = 0.026). C Reticulocyte production (Friedman; BALB/c: χ2 = 10, df = 3, P = 0.019; C57BL/6: χ2 = 9.8, df = 3, P = 0.020). #P < 0.05, ##P < 0.01, ###P < 0.001: values are significantly different between host strains. [file 13071_2024_6286_MOESM4_ESM.jpg]

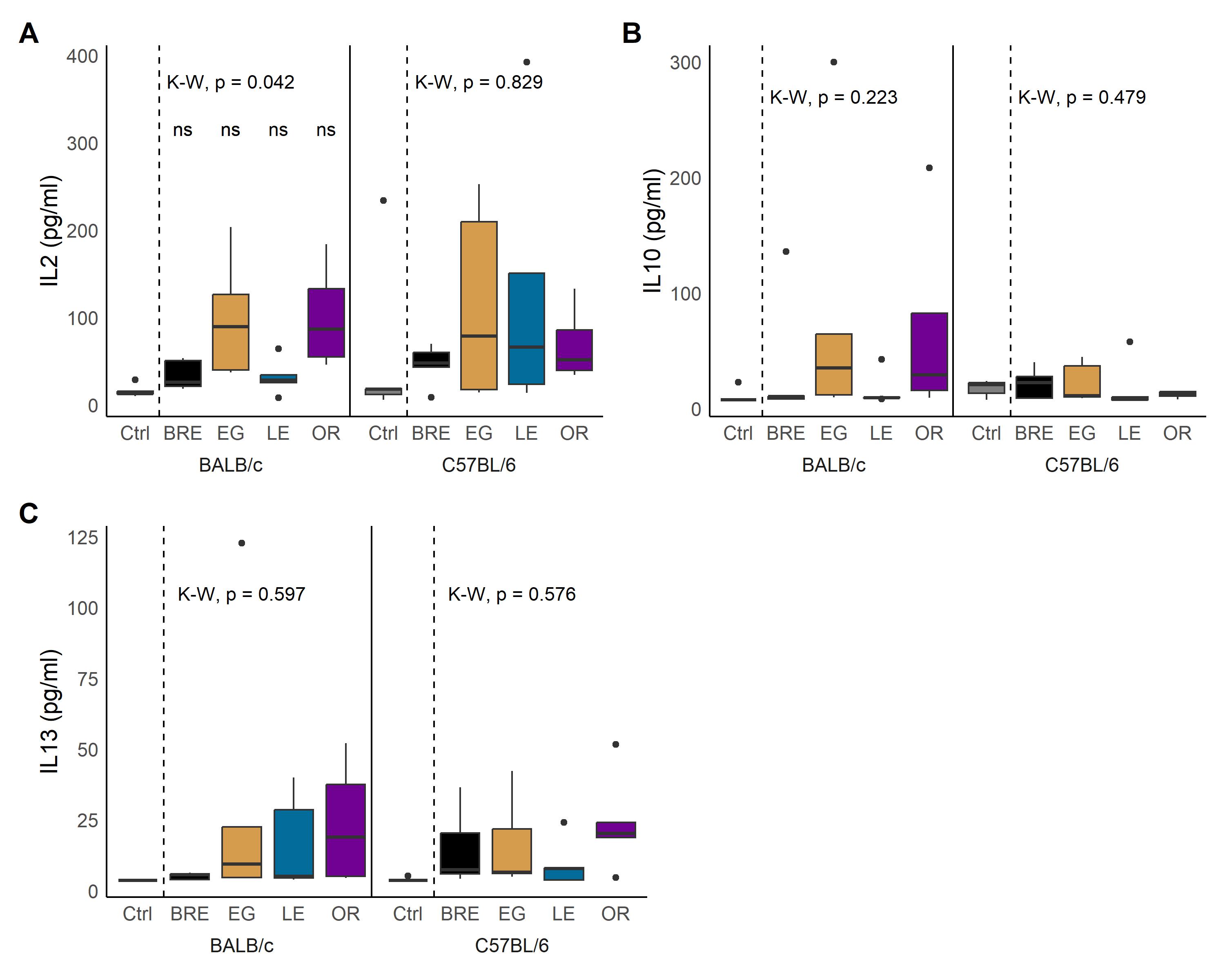

Supplement: Supplementary file 5 — Additional file 5: Figure S4: No differences in IL-2, IL-10 and IL-13 levels. A Despite a significant Kruskal-Wallis test in BALB/c mice (Kruskal-Wallis; BALB/c: H = 8.2, df = 3, P = 0.042; C57BL/6: H = 0.886, df = 3, P = 0.829), IL-2 secretion was not significantly different between parasite populations as analyzed by a Dunn post hoc test. B IL-10 (Kruskal-Wallis; BALB/c: H = 4.39, df = 3, P = 0.223; C57BL/6: H = 2.48, df = 3, P = 0.479) and C IL-13 (Kruskal-Wallis; BALB/c: H = 1.88, df = 3, P = 0.597; C57BL/6: H = 1.98, df = 3, P = 0.576) levels were not significantly different between parasite populations or mouse lines. K-W = Kruskal-Wallis. [file 13071_2024_6286_MOESM5_ESM.jpg]

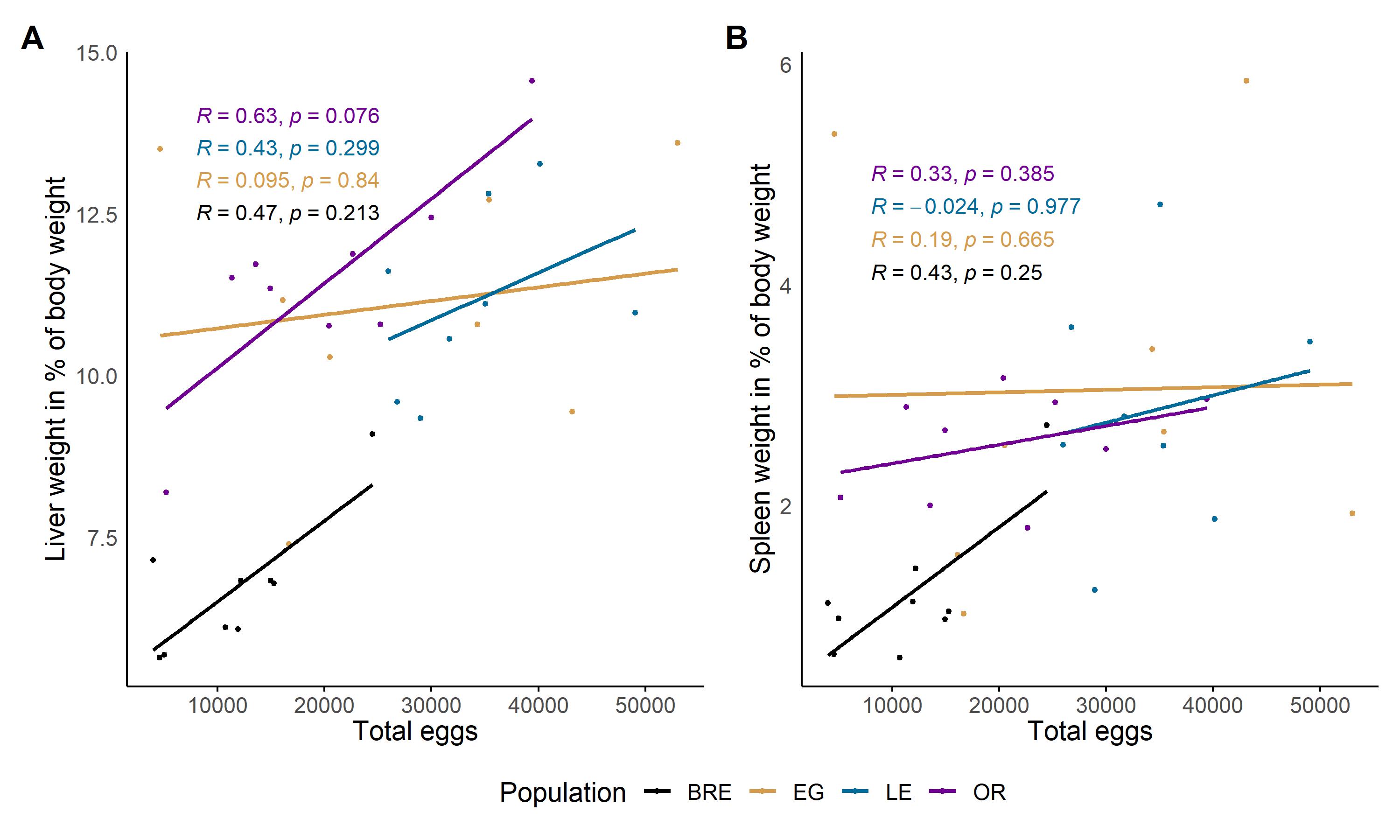

Supplement: Supplementary file 6 — Additional file 6: Figure S5: Correlation plots between total egg counts and liver/spleen weight. A Normalized liver weight is not significantly correlated in any of the parasite populations (Spearman’s correlation coefficient; BRE: rs = 0.47, P = 0.213, EG: rs = 0.095, P = 0.840, LE: rs = 0.43, P = 0.299, OR: rs = 0.63, P = 0.076). B Normalized spleen weight does not significantly correlate with total egg burden in the four parasite populations examined (Spearman’s correlation coefficient; BRE: rs = 0.43, P = 0.25, EG: rs = 0.19, P = 0.665, LE: rs = -0.024, P = 0.977, OR: rs = 0.33, P = 0.385). [file 13071_2024_6286_MOESM6_ESM.jpg]
